# Individual Effect Modifiers of Dust Exposure Effect on Cardiovascular Morbidity

**DOI:** 10.1371/journal.pone.0137714

**Published:** 2015-09-18

**Authors:** Alina Vodonos, Michael Friger, Itzhak Katra, Helena Krasnov, Doron Zahger, Joel Schwartz, Victor Novack

**Affiliations:** 1 Department of Public Health, Ben-Gurion University of the Negev, Be’er-Sheva, Israel; 2 Clinical Research Center, Soroka University Medical Center, Be’er-Sheva, Israel; 3 Department of Geography and Environmental Development, Ben-Gurion University of the Negev, Be’er-Sheva, Israel; 4 Intensive Cardiac Care Unit, Department of Cardiology, Soroka University Medical Center, Be’er-Sheva, Israel; 5 Department of Environmental Health, Harvard School of Public Health, Boston, Massachusetts, United States of America; The Ohio State University, UNITED STATES

## Abstract

**Background:**

High concentrations of particulate matter (PM) air pollution have been associated with death and hospital admissions due to cardiovascular morbidity. However, it is not clear a) whether high levels of non-anthropogenic PM from dust storms constitute a health risk; and b) whether these health risks are exacerbated in a particular demographic.

**Methods:**

This study comprised all patients above 18 years old admitted to Soroka University Medical Center (1000 bed tertiary hospital, Be’er- Sheva, Israel, 2001–2010) with a primary diagnosis of acute coronary syndrome (ACS). Data on meteorological parameters and PM_10_ (particulate matter <10 μm in aerodiameter) were obtained from monitoring stations in the city of Be'er-Sheva. Data were analyzed using a case crossover analysis to examine the effect of dust exposure on hospitalization due to ACS and the interaction with co-morbidities and demographic factors.

**Results:**

There were 16,734 hospitalizations due to ACS during the study period. The estimated odds of hospitalization due to ACS was significantly associated with PM_10_ during non dust storm days at the same day of the exposure (lag0); OR = 1.014 (95%CI 1.001–1.027) for a 10 μg/m^3^ increase, while a delayed response (lag1) was found during the dust storm days; OR = 1.007 (95%CI 1.002–1.012). The effect size for the dust exposure association was larger for older (above the age of 65), female or Bedouin patients.

**Conclusions:**

Exposure to non-anthropogenic PM is associated with cardiovascular morbidity. Health risk associated dust exposure is gender and age specific with older women and Bedouin patients being the most vulnerable groups.

## Introduction

High concentrations of particulate matter (PM) air pollution are associated with mortality and hospital admissions for cardiovascular causes.[1–6]

Population subgroups might vary in their response to particulate air pollution. Bateson and Schwartz [7] showed that the risk of death associated with PM_10_ (particulate matter <10 μm in aerodiameter) exposure appeared to decrease with age among men and increase with age among women. Other studies have suggested that the elderly [1,8] and those with less than a high school education (low socioeconomic status) may be particularly susceptible.[9] A study performed in Chicago [10] showed that the air-pollution-associated increase in hospital admissions for cardiovascular diseases was almost doubled in subjects with concurrent respiratory infections, suggesting that patients with acute respiratory infections are a risk group for particulate matter effects. The same study also reported a higher effect of PM_10_ on CVD admission in females compared to males and in those older than 75 years of age compared to younger persons.

Recently, special attention has been given to non- anthropogenic air pollution originating from natural dust storms, which may constitute a health risk threat.[2,11–12] The annual global dust loading from soils into the atmosphere is estimated to be as much as 5 billion ton.[13] The Negev region of Israel is located between the Saharan and the Arabian deserts (the world’s largest dust-belt) and is exposed every year to several intense dust storms. During dust storms in this region, PM levels can significantly exceed those defined as acceptable in terms of air quality and human health (50 μg/m^3^) with hourly concentrations of 100–5000 μg/m^3^.[14] During recent decades, there is a trend of increasing dust storm events frequency and intensity in the Mediterranean basin.[15] This trend may continue due to droughts and intense soil uses in Europe and Mediterranean, which lead to increased soil erodibility and dust emission.

We sought to determine whether high levels of PM from dust storms constitute a cardiovascular health hazard and what individual characteristics might modify the potential health effect.

## Materials and Methods

### Population

We conducted a retrospective cohort study comprising adult patients admitted to Soroka University Medical Center (SUMC) with a primary diagnosis of acute coronary syndrome (ACS); (ICD-9 codes 411.81(ACS), 411.1(unstable angina, UA), 410.9,410 (acute myocardial infarction (AMI), non ST elevation myocardial infarction (NSTEMI), ST elevation myocardial infarction (STEMI)) between 2001 and 2010.

SUMC is the only medical center in this area serving a population of approximately 700,000 as the primary hospital and nearly 1 million as a tertiary hospital. The study area is inhabited by two main ethnic groups: predominantly urban Jews (69%) and rural Bedouin Arabs (31%).

The following patient level data were obtained using the centralized electronic medical records database: diabetes (ICD-9 code 250), history of MI (ICD-9 code 410–414), hypertension (ICD-9 code), chronic obstructive lung disease (COPD) (ICD-9 code 490, 491, 492, 496) and socio-demographic data such as gender, age and ethnicity (Jews vs. Bedouin Arabs).

#### Ethics Statement

The study was approved by the institutional review board of Soroka University Medical Center. Patient information was anonymized and de-identified prior to analysis, therefore written informed consent was not obtained.

### Environmental Data

Data for the study period included PM_10_ concentrations (μg/ m^3^), NO_2_ (μg/m^3)^, meteorological parameters (temperature (C^O^) and relative humidity (%)) obtained from monitor station in Be'er-Sheva operated by the Ministry of Environmental Protection. This is the only monitoring station in the area recording all pollutants simultaneously in 20 minute intervals. The monitoring station is located in a typical neighborhood in the center of Be’er-Sheba (the city area is about 20 km^2^). Thus, we have limited our population to reside within 20km radius. Seasons were defined according to Alpert et al.:[16] winter (December 7-March 30), summer (May 31-September 22) each last about 4 months (3 months and 23 days), autumn (September 23-December 6) and spring (March 31-May 30) each lasts only 2 months (75 days and 61 days, respectively).

### Dust Storm definition

The primary source of PM_10_ pollution in the region is non-anthropogenic. Most of the dust storms in the Negev region originate from the North Africa. Strong dust storms can last from several hours to few days. The daily average concentration of atmospheric PM_10_ can reach more than 2000 μg/ m^3^, with hourly concentrations as high as 5000 μg m^3^. We defined a dust storm (DS) day as a day with an averaged PM_10_ concentration that was two standard deviations (SD) above the background value. [14]The background value was calculated on the basis of the average PM_10_ concentration for 12-hour period (from 6 am to 6 pm) during the summer season (dust free period). It reflected the background level inclusive of non-anthropogenic and anthropogenic sourced particles.[17] Furthermore, we have validated the definition of dust storm by examination in details of the back-trajectories and synoptic conditions during the dust day to confirm that the origins of the dust were external to the region. The resulting threshold value for a dust day was 71 μg/ m^3^.[14]

### Statistical analysis

The statistical analysis involved two steps. First, generalized additive Poisson regression models (GAM) were used to test a possible non-linear association of PM_10_ on incidence of ACS. To adjust for seasons and other temporal effects in GAM models, we used a non-linear terms with penalized splines for time with five degrees of freedom per year. Similarly, we used the same approach with five degrees of freedom for average temperature to adjust for a possible non-linear effect of temperature. The aim of the second step was to test the modification of the effect of PM_10_ on the ACS risk by different co morbidities (diabetes, history of MI, hypertension, COPD) and demographic factors (age and gender and ethnicity). This analysis followed a case crossover procedure where each subject with event contributes information as a case and as matched control during nonevents times.[18]

We used two different methods of case definitions (exposure period); (1) case period was defined as the hospitalization day, while for the control period we used every third day either before or after the hospitalization day within the same calendar month [19] (2) case and control period defined as fix periods of 8 weeks strata (a month from a hospitalization day, bidirectional). The case period defined as three days mean of pollution ending on the hospitalization day (lag 0–2). The control periods were chosen to end on the same week-day as a hospitalization day during the weeks before and after the hospitalization (four before and four after the case period). For example, if the hospitalization occurred on Monday; the three day’s mean PM_10_ concentrations were compared to eight similar periods on preceding and following Mondays. Both strategies belong to symmetric bidirectional case crossover design where control periods are symmetrically spaced in time minimizing the potential time-varying confounders by season or secular trends.[20]

Conditional logistic regression models were conducted to analyze the exposure odds ratio (ORs) with 95% confidence intervals (CI) on risk of ACS hospitalization. To investigate the potential modification of the effect of PM_10_ on the ACS risk by different co morbidities and demographic factors, we simultaneously added to the model sets of 2-way and 3-way interaction terms that expressed the impact of PM_10_ measurement among vulnerable population (demographic and clinical personal characteristics; diabetes, history of MI, hypertension, COPD, gender, age and ethnicity (Jews vs. Bedouin Arabs)). We controlled for daily mean temperature and humidity, indicator of the day of the week for the first case definition method and an indicator variable of month (1; 31) additionally included in the models based on the second case definition method. We performed a stratified analysis to compare the magnitude of the effect among different patients with STEMI (ST elevation myocardial infarction) and NSTEMI (non ST-elevation myocardial infarction (NSTEMI). Analyses were performed using R statistical software, version 3.0.

## Results

### Patient population

The study population comprised 12,177 patients with 16,734 hospitalizations due to ACS. **Table 1**depicts study population demographics and frequent co-morbidities. Overall 5,500 admissions were due to STEMI (ST elevation myocardial infarction) and 5,261 were NSTEMI (non ST-elevation myocardial infarction (NSTEMI), the remaining patients were diagnosed with unstable angina (34.3%).The average age of ACS patients was 65.8±13.3years with 54.2% above the age of 65. The majority (68.7%) were male and 10.5% were Bedouin Arabs. Frequent co-morbidities included diabetes mellitus (DM) (30.1%), hypertension (50.1%), congestive heart failure (6.4%) and history of MI (23.1%). Female patients were older then male (71.6±1 vs. 63.1±1), and had higher rate of DM (38.8% vs. 26.3%), hypertension (65.1% vs. 43.2%), but not history of MI (19.9% vs. 24.8%) or COPD (4.2% vs. 7.1%). Bedouin Arabs patients, compared to the Jewish patients had a higher rates of DM (34.8% vs. 29.8%) and COPD (13.9% vs. 5.3%), but not hypertension or history of MI (43.8% vs. 51.1% and 23.1% vs. 24.7%).

**Table 1:** Patient characteristics of study population during study period 2001–2010 (N = 16,734).

Number of ACS Cases	Mean ± SD or N (%)
STEMI	5,500 (31.4%)
NSTEMI	5,261 (32.8%)
Unstable Angina	5,826 (34.3%)
**Gender**	
Male	11,458 (68.5%)
Female	5,276 (31.5%)
**Bedouin Arabs**	1,762 (10.53%)
**Age, Mean±SD**	65.8±13.3
Age >65	9,062 (54.15%)
**Frequent co morbidities**	
Diabetes Mellitus	5,054 (30.08%)
Hypertension	8,383 (50.10%)
History of MI	3,871 (23.13%)
COPD	1,045 (6.24%)
Hospital mortality	697 (4.2%)
Total Mortality (in and out hospital)	5,558 (33.12%)

### Dust exposure

Daily average concentrations of PM_10_, NO_2_, as well as daily meteorological data are described in **Table 2**.Overall, the PM_10_ levels exceeded the WHO recommended daily threshold of 50 μg/m^3^[21]during 955 (26.15%) days of the study period. Based on the definition of a dust storm (levels above 71μg/m^3^), we have identified 445 (12.2%) dust storm days with the majority occurring during the winter and spring seasons (from December to May). The levels of NO_2_ (μg/m^3^) during all days were 19.2±15.2, for dust storm days = 20.1±16.3 and for non-dust storm days = 19.2±15.0. During the "rush hour" (8:00 am) the levels of NO_2_ were slightly higher reaching 24.8±14.8 μg/m^3^.

**Table 2:** Descriptive statistics for PM_10_ exposure and Meteorological factors in Southern Israel 2001–2010.

Covariate	Summer	Autumn	Winter	Spring
	(May 31-Sep 22)	(Sep 23-Dec 6)	(Dec 7- Mar 30)	(Mar 31-May 30)
Mean PM_10_(mg/μ^3^)	40.4±17.1	49.3±44.2	67.9±137.7	69.2±122.9
Maximum PM_10_(mg/μ^3^)	96.8±43.2	118.2±81.9	186.6±157.9	162.3±148.2
Mean PM_10_(mg/μ^3^) in dust storm days	852	1107	3873	4797
Num. Dust storm days, n(%)	37 (3.2%)	98 (13.1%)	195 (17.1%)	115 (18.9%)
Mean NO_2_ (mg/μ^3^)	13.7±5.1	22.7±9.5	22.7±11.1	15.9±6.4
Mean Temperature (°C)	25.8±1.8	20.5±3.7	13.6±3.4	20.4±3.8
Maximum Temperature (°C)	31.9±2.7	25.9±4.9	18.7±4.8	26.9±5.2
Relative Humidity (%)	69.4±10.9	67.6±18.7	69.6±18.7	60.8±17.1

### Effect of PM_10_ and dust storm days on risk of hospitalization due to ACS

**Fig 1:**
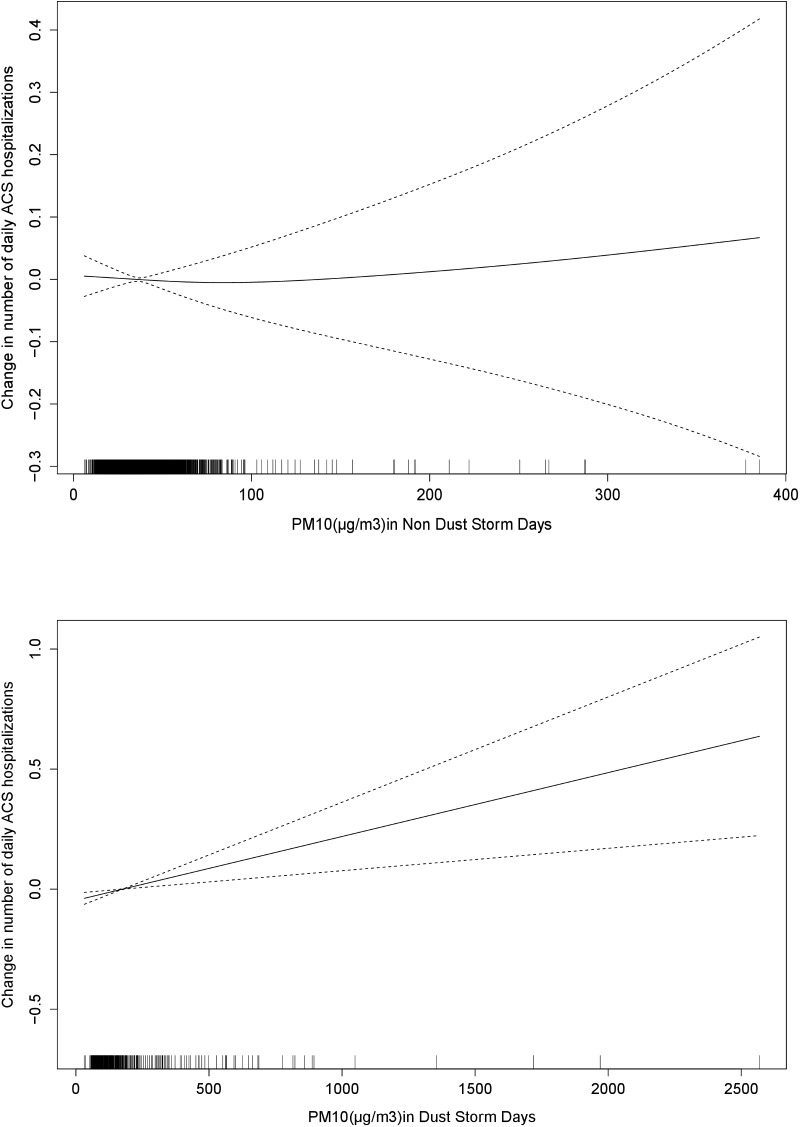
Change in number of daily ACS hospitalizations associated with increase in PM_10_ (μg/m^3^), during non dust and dust storm days. The solid line represents the change in number of daily ACS hospitalizations associated with increase in PM_10_ (μg/m^3^), the dotted line indicates the 95%CI (confidence interval).

The results of the Poisson based model analysis showed that the impact of PM_10_ concentration on ACS incidence differed between dust storm and non-dust storm days (**Fig 1**). The estimated RR for dust storm day (yes/no variable) was 1.051 (95%CI 1.005–1.100) at the same day of the hospitalization (lag 0) and 1.024 (95%CI 0.977–1.071) at lag1. Results based on the conditional logistic regression model showed that theOR for ACS associated with a 10 μg/m^3^ increase in the concentration of PM_10_ during non dust storm days was 1.014 (95%CI 1.001–1.027), compared with 1.001 (95%CI 0.995–1.007) during the dust storm days (hospitalization day exposure, lag 0). Conversely, one day delayed PM_10_ effect (lag 1) following the dust storm days was associated with hospitalization due to ACS; OR = 1.007 (95%CI 1.002–1.012), while there was no significant effect during non dust storm days (OR = 1.011, 95%CI 0.998–1.025) (**Table 3**). A cumulative effect of 3 days (lag 0–2) showed a significant association between PM during the dust storm days: OR for ACS hospitalizations of 1.004 (95%CI 1.000–1.008). This effect of a cumulative exposure was season dependent being higher during winter (OR = 1.007 95%CI 1.002–1.012), as compared to summer, autumn or spring season, OR = 1.002 (95% CI 0.986–1.019), OR = 1.005 (95% CI 0.989–1.020) and OR = 0.996 (95% CI 0.986–1.007) respectively.

**Table 3:** OR^*^ for hospital admission due to ACS associated with increase in 10 μg/m^3^ of PM_10_ in dust storm days, and non-dust storm days.

		Main effect	PM*Female	PM*Female*Age65	PM*Age65*Bedouin
**lag 0**	PM_10_^all^	1.002 (0.996–1.008)	0.998 (0.985–1.011)	**1.015 (1.002–1.027)**	**1.023 (1.003–1.043)**
	PM_10_^nd^	**1.014 (1.001–1.027)**	0.770 (0.941–1.008)	1.029 (0.997–1.063)	1.021 (0.972–1.072)
	PM_10_^d^	1.001(0.995–1.007)	0.999 (0.986–1.008)	**1.014 (1.002–1.027)**	**1.021 (1.002–1.042)**
**lag 1**	PM_10_^all^	1.003 (0.998–1.009)	1.002 (0.991–1.014)	1.006 (0.995–1.017)	**1.022 (1.009–1.037)**
	PM_10_^nd^	1.011 (0.998–1.025)	0.980 (0.948–1.012)	1.016 (0.985–1.047)	0.997 (0.951–1.044)
	PM_10_^d^	**1.007(1.002–1.012)**	0.997 (0.985–1.012)	1.005(0.994–1.016)	1.024 (1.010–1.040)
**lag 2**	PM_10_^all^	1.004 (0.998–1.010)	0.993 (0.980–1.006)	1.006 (0.993–1.018)	1.013 (0.998–1.030)
	PM_10_^nd^	1.015(0.999–1.030)	0.993 (0.963–1.025)	1.006 (0.973–1.039)	1.003 (0.954–1.054)
	PM_10_^d^	1.004(0.998–1.010)	0.993 (0.980–1.025)	1.006 (0.994–1.019)	1.013 (0.997–1.029)
**lag 0–2**	PM_10_^all^	1.004 (1.000–1.001)	0.998 (0.990–1.006)	1.005 (0.997–1.013)	**1.019 (1.031–1.008)**
	PM_10_^nd^	1.006 (0.998–1.015)	0.991 (0.973–1.009)	1.002 (0.984–1.020)	1.018 (0.992–1.045)
	PM_10_^d^	**1.004 (1.000–1.008)**	0.998 (0.988–1.006)	1.005 (0.997–1.013)	**1.020 (1.008–1.031)**

PM_10_^all^–PM_10_ in all days

PM_10_^nd^—PM_10_ in non-dust storm days

PM_10_^d^ - PM_10_ in dust storm days

Boldface indicates positive statistical significant results (p-value <0.05)

*Odds Ratio and 95%CI of hospital admission for ACS associated with increase in 10μg/m^3^ PM_10,_ results of conditional logistic regression adjusted for interaction with demographic and clinical personal characteristics, temperature, relative humidity, day of the week and month.

We extended the basic model to include interaction terms representing the independent interaction between PM_10_ concentrations and each of the medical conditions of interest: diabetes, history of MI, hypertension and COPD and demographic characteristics; gender, age and ethnicity (Jews vs. Bedouin Arabs).An increase in 10 μg/m^3^ in PM_10_ concentration during dust storm days was associated with ACS hospitalization risk among older women (> 65 years); OR = 1.014 (1.002–1.027) compared to the effect during non dust storm days (OR = 1.029 95%CI 0.997–1.063). In addition, a strong effect was found among the older Bedouin population, during the dust storm days; OR = 1.021 (95%CI 1.002–1.042) compared to the effect during non dust storm days (OR = 1.021 95%CI 0.972–1.072) (Table 3). This risk was particularly high in the latter group during winter season: OR = 1.025 (95% CI 1.010–1.040).

**Fig 2:**
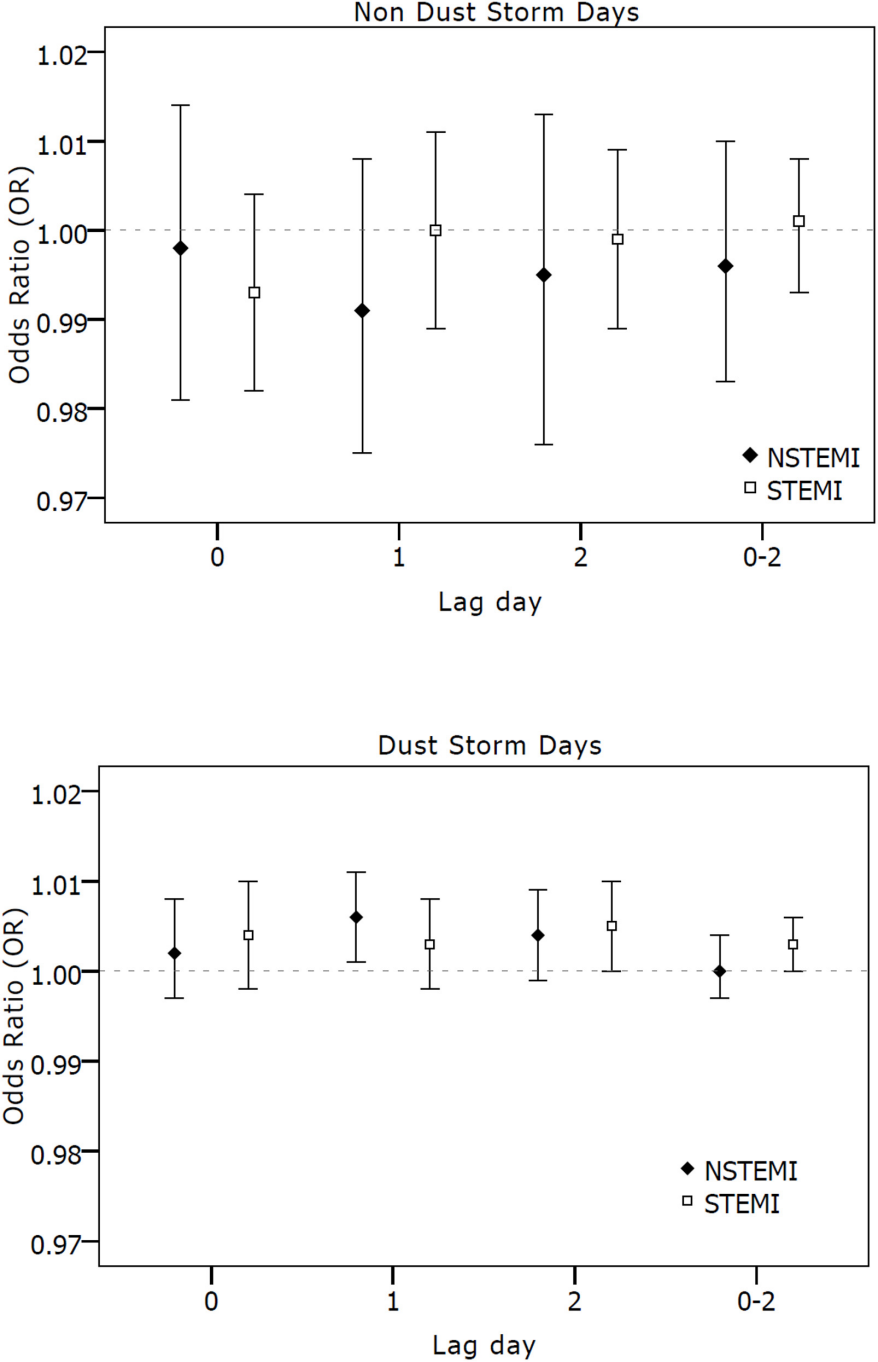
OR for hospital admission due to NSTEMI and STEMI for 10μg/m^3^ in short term PM_10_ during non dust (a) and dust storm days (b). OR for hospital admission due to NSTEMI and STEMI for 10μg/m^3^ in short term PM_10._ Error bars indicate 95% CI (confidence interval).

In addition to the age, gender and ethnicity effect, we found that older patients with a history of a prior MI were at risk for hospitalization due to ACS at the day of the exposure during the dust storm days: OR of 1.014 per 10 μg/m^3^ increase (95%CI 1.001–1.028) compared to patients without a history of MI (OR = 0.991, 95%CI 0.979–1.003). We did not find interactions with diabetes and COPD. Stratified analysis for non-ST elevation myocardial infarction (NSTEMI) and ST elevation myocardial infarction (STEMI) (**Fig 2**) did not show PM_10_effect at hospitalization day (lag0), both for dust storm and non dust storm days. Exposure at the day preceding hospitalization (lag1) was associated with a higher risk among the NSTEMI group compared to the STEMI during the dust storm days; OR of 1.006 (95%CI 1.001–1.011) and OR of 1.003 (95%CI 0.998–1.008), respectively. In contrast, two days prior to the hospitalization (lag 2) exposure effect in STEMI group was higher as compared to the NSTEMI group; OR = 1.005 (95%CI 1.000–1.010) and OR = 1.004 (95%CI 0.999–1.009), respectively. No difference was found between the groups during the non-dust storm days.

## Discussion

In this population based study we aimed to investigate the association between particular matter exposure and cardiovascular morbidity and to identify individual characteristics predisposing for a negative health effect. We have shown an association between particulate air pollution and the incidence of hospitalizations with acute coronary syndrome. The exposure effect varied with gender, age and ethnicity. The most vulnerable group were Bedouin Arabs over the age of 65 for both immediate and “lag” effects.

Current evidence suggests that the effect of a short term increase in PM exposure on cardio-pulmonary morbidity and mortality may be mediated through pulmonary inflammation causing increased blood coagulability, abundance of endothelial adhesion molecules, precipitating acute coronary syndromes. In addition alterations in vascular tone and endothelial function contribute to ischemic event risk.[22–24]

The majority of the environmental research analyzing the effects of air pollution on cardiovascular health focuses on anthropogenic air pollution (e.g. transport, industry etc). In the current study we analyzed in addition to the "regular" exposure, the association between dust exposure and acute coronary syndrome hospitalization for dust storm days during which a high PM concentration is due to the natural phenomenon. The immediate association was found during non dust storm days, while a two days lagged effect was found during the dust storm days. Our findings are comparable to the ten years analysis of cardiovascular morbidity in Nicosia Cyprus [12], which showed that cardiovascular admissions were 10.4% (95%CI, -4.70025–27.9%) higher during dust storm days. Studies from the Caribbean, Europe and Israel showed that PM pollutants contained in the Sahara dust increased the risk of Asthma emergency department visits among children [25, 26], cardiac and respiratory hospital admissions [27,28] and mortality [29].However, Sajaniet. al[30]did not find evidence of an effect modification of dust events on the relationship between PM_10_ and health outcome (daily death). Furthermore a study conducted in Greece [31] showed a greater effect of PM_10_ on mortality during days with high PM exposure due to anthropogenic sources as compared to days with high desert dust originated PM exposure. The risk increment of hospitalizations due to ACS attributed by the dust exposure that were found in our current study is relatively small, even smaller then in our previous study, were we reported an increase of hospitalizations due to COPD exacerbation by 17% [32]. We hypothesize that traffic and industry related particles have more toxic effects than non-anthropogenic sources from Sahara dust on human health [31]. The Negev region is a semi-arid environment, thus the primary source of PM_10_ pollution is non-anthropogenic as confirmed by low levels of NO_2_ (traffic related pollutant) found during the dust storm days. Therefore, we believe that in large cities like Rome, Madrid and Athens have higher concentration of anthropogenic components in PM_10_ presumably being more biologically active. Therefore, the predominance of the anthropogenic components in PM_10_ in other areas can explain the difference in risk estimates between those cities and our region.

The current analysis showed that older (above the age of 65) females or Bedouin Arabs patients were at the highest risk for hospitalization due to ACS following OM exposure. These findings are consisted with several other studies [7,10,33] showing that female and older patients are at higher risk of death associated with PM_10_ exposure. Chen et. al. also found a positive association between fatal coronary heart disease and particulate air pollution exposure in females, but not in males. [34] Zanobetti et al. [10] observed a significant effect of PM_10_ on CVD admission for females and elderly (above 75 years), but not in males or younger subjects.

The higher PM effect in elderly women observed in our study can be explained by epidemiological and biological mechanisms. The sex and age differences could also reflect a measurement error in the assigned exposure due to differences in time spent in commuting and location of work places between men and women and between young and elderly patients. Empirical studies on mobility suggest women have smaller activity spaces than men and younger, meaning they tend to spend more time in and around the household. [35] The same is probably true of the elderly compared with younger patients. This exposure measurement error in males and younger subjects may result in bias toward zero, i.e. type II error. [36].We also hypothesized that the observed interaction between gender and PM effect may reflect biologic causes. Differing particulate deposition patterns between men and women may partially explain this difference in the effect.[37]Another potential explanation of the interaction with both age and gender is that premenopausal women are naturally protected against atherosclerosis by endogenous hormones, while loss of hormonal protection would lead to increased vulnerability after menopause. [38]

We did not find a significant interaction of the PM effect with a history previous admissions due to respiratory conditions such as chronic obstructive pulmonary disease [10]or diabetes[7] observed in previous studies.

The study area is inhabited by two main ethnic groups: predominantly urban Jews (69%) and rural Bedouin Arabs (31%). These two ethnic groups differ greatly in their socioeconomic and cultural background, as well as in their living environment. The majority of the Jewish population lives in the city of Be'er-Sheva, towns and agricultural settlements, whereas the Bedouin population lives mostly in rural settlements with a significant portion of the Bedouin population resides in shacks and tents [39] making them more exposed to outdoor dust. We hypothesize that these living conditions explain the study finding of older Bedouin population being particularly susceptible to the dust exposure effect.

The individual increase in cardiovascular risk due to air pollution as found in our study is relatively small compared to the impact of conventional cardiovascular risk factors (e.g. diabetes). However, as the dust exposure involves the whole population, even a subtle increase in risk represents a public health concern and may have implications on public health policies.[40] Another important aspect to consider is the time scale of the exposure. The magnitude of the adverse health effect depends on both the potency and the duration of exposure. [22,41] Therefore despite the small effect found for the majority of the environmental pollutants, long term repeated exposures to pollution may have a significant health impact.

The strengths of this study rely on the fact that it is based in a single tertiary hospital which serves all population in the region, thus eliminating selection bias. The exposure analysed in our study is of non-anthropogenic nature with most of the dust storm originate from the North Africa. The unique combination of a centralized modern medical system and urban population residing in this arid and hot region makes the Negev an ideal "environmental laboratory" for studying the health effect of global environmental change such as desertification and global warming.

Our study has several important limitations. We used outdoor air pollution concentrations measured at fixed point monitors, whereas people spend most of the time indoors. The obtained assessments, based on the information from fixed monitor stations, might have resulted in a non-differential exposure bias, which might have reduced the magnitude of the association toward the null hypothesis and produced a wider confidence intervals due to a mixture of classical and Berkson error. [42]Furthermore, our group has previously shown a significant contribution of dust events to the indoor PM_2.5_ and PM_10_ levels, in which the PM_10_concentrations at houses can reach even 1000 μg/m^3^ for several hours.[43]

In conclusion, exposure to non-anthropogenic particular matter is associated with an increased risk of acute coronary syndrome. It appears that health risk is gender, age and ethnicity specific with older women or Bedouin Arabs patients being a particularly vulnerable group. Despite the relatively low effect size, its clinical significance is due to the prolonged dust exposure in the entire population.Further investigation is needed to understand the mechanism of the effect and to identify additional vulnerable population groups.
